# Functional characterization of human equilibrative nucleoside transporter 1

**DOI:** 10.1007/s13238-016-0350-x

**Published:** 2016-12-19

**Authors:** Weiyun Huang, Xin Zeng, Yigong Shi, Minhao Liu

**Affiliations:** Beijing Advanced Innovation Center for Structural Biology, Tsinghua-Peking Joint Center for Life Sciences, School of Life Sciences, Tsinghua University, Beijing, 100084 China

**Keywords:** hENT1, nucleoside, functional characterization, transport, counter flow

## Abstract

Equilibrative nucleoside transporters (ENTs), which facilitate cross-membrane transport of nucleosides and nucleoside-derived drugs, play an important role in the salvage pathways of nucleotide synthesis, cancer chemotherapy, and treatment for virus infections. Functional characterization of ENTs at the molecular level remains technically challenging and hence scant. In this study, we report successful purification and biochemical characterization of human equilibrative nucleoside transporter 1 (hENT1) *in vitro*. The HEK293F-derived, recombinant hENT1 is homogenous and functionally active in proteoliposome-based counter flow assays. hENT1 transports the substrate adenosine with a K_m_ of 215 ± 34 µmol/L and a V_max_ of 578 ± 23.4 nmol mg^−1^ min^−1^. Adenosine uptake by hENT1 is competitively inhibited by nitrobenzylmercaptopurine ribonucleoside (NBMPR), nucleosides, deoxynucleosides, and nucleoside-derived anti-cancer and anti-viral drugs. Binding of hENT1 to adenosine, deoxyadenosine, and adenine by isothermal titration calorimetry is in general agreement with results of the competitive inhibition assays. These results validate hENT1 as a bona fide target for potential drug target and serve as a useful basis for future biophysical and structural studies.

**Electronic supplementary material:**

The online version of this article (doi:10.1007/s13238-016-0350-x) contains supplementary material, which is available to authorized users.

## Introduction

Uptake of nucleoside is required for DNA and RNA synthesis and plays a key role in signaling, exemplified by adenosine interaction with the cell surface P1 purinergic receptors, which elicits a range of physiological responses including coronary vasodilation, renal vasoconstriction, and platelet aggregation (Kong et al., 2004; Jaakola et al., 2008; Young et al., 2013; Wall and Richardson, 2015). The same mechanism that mediates nucleoside uptake is also responsible for the transport of nucleoside-derived drugs, which are widely used to treat virus infections and cancer (King et al., 2006; Jordheim and Dumontet, 2007; Jordheim et al., 2013). In mammalian cells, nucleosides are transported by two different nucleoside transporters: the concentrative sodium-dependent nucleoside transporter (CNT), which corresponds to the SLC28 family, and the equilibrative nucleoside transporter (ENT), which pertains to the SLC29 family (Baldwin et al., 2004; Gray et al., 2004; Young et al., 2013; Young, 2016).

The ENT family, identified in many higher eukaryotes, contain four members in human, namely hENT1, hENT2, hENT3 and hENT4 (Griffiths et al., 1997a; Griffiths et al., 1997b; Hyde et al., 2001; Baldwin et al., 2004; Barnes et al., 2006; Govindarajan et al., 2009; Boswell-Casteel and Hays, 2016; Young, 2016). hENT1 shares 47, 33, and 24 percent sequence identity with hENT2, hENT3, and hENT4, respectively. All ENTs are thought to share the same general topology and structure, which consist of 11 transmembrane segments (TMs), a cytoplasmic N-terminus and an extracellular C-terminus, a large extracellular loop between TM1 and TM2, and a large cytoplasmic loop between TM6 and TM7 (Sundaram et al., 2001; Valdes et al., 2009; Valdes et al., 2014).

Expression of ENTs is a predictive biomarker for drug efficacy in cancer treatment (Giovannetti et al., 2006; Borbath et al., 2012; Nordh et al., 2014; Pastor-Anglada and Perez-Torras, 2015; Svrcek et al., 2015). However, the current level of knowledge on the structure and function of ENTs is vastly inadequate, given their general importance in drug development. There is no detailed structural information on any member of the ENT family. Rigorous effort aimed at structural elucidation of ENTs has been hampered by the technical challenge of recombinant expression in functional form. At present, the only piece of relevant structural information is the crystal structure of CNT transporters (CNTs) from Gram-negative bacterium *Vibrio cholera* (vcCNT) (Johnson et al., 2012; Johnson et al., 2014). vcCNT shares 14.9 percent sequence identity with hENT1. Although this information facilitates our understanding of the molecular mechanism for nucleoside transport and recognition of the CNTs, it provides no assistance to mechanistic understanding of the ENTs. Therefore, hENT1 represents a highly prioritized target for biochemical and structural investigation.

In this study, we report successful overexpression and purification of hENT1 in human HEK293F cells and characterization of its biochemical and biophysical properties. We reconstituted a substrate transport assay using hENT1-incorporated proteoliposomes for *in vitro* functional studies. We report the kinetics and transport activities of hENT1 for a number of nucleosides and nucleoside-derived drugs in proteoliposome-based counter flow assays. This information constitutes an important basis for mechanistic understanding of hENT1 as well as for future structural studies.

## Results

### Expression and purification of hENT1

**Figure 1:**
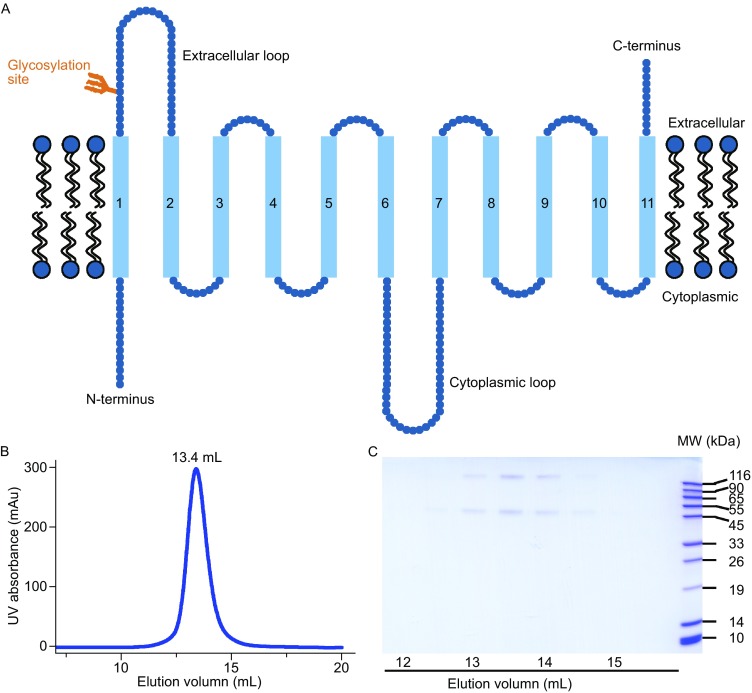
Expression and purification of hENT1 in HEK293F cells. (A) The predicted topology diagram of hENT1. hENT1 has 11 transmembrane segments, with a cytoplasmic amino-terminus and an extracellular carboxyl-terminus. The positions of the glycosylation site and two long loops are indicated. (B) A representative chromatogram of hENT1 from gel filtration chromatography. The elution volume of hENT1 in the Superdex-200 column gel filtration is indicated. (C) Visualization of hENT1 on a SDS-PAGE gel. The peak fractions from gel filtration were applied onto a SDS-PAGE gel followed by coomassie blue staining

The predicted secondary structure of hENT1 (residues 1–456) conforms to that of the SLC29 family, with 11 TMs, an amino-terminus in the cytoplasm, and a carboxyl-terminus in the extracellular side (Fig. 1A). hENT1 contains two extended loops: one between TM1 and TM2, and the other between TM6 and TM7 (Fig. 1A). A known N-linked glycosylation site on Asn48 is located in loop 1 (Sundaram et al., 2001) (Fig. 1A). During intense effort in the past several years (Li et al., 2009; Reyes et al., 2010; Reyes et al., 2011; Rehan et al., 2015; Rehan and Jaakola, 2015), none of the four hENTs has been purified to homogeneity and consequently no purified hENT has been biochemically characterized. To obtain recombinant, homogeneous hENT1 for functional studies, we explored different expression systems—bacterial, baculovirus-infected insect cells, and mammalian cell tissue culture—in combination with exhaustive protein engineering endeavor. After years of trials, we focused on the mammalian expression system.

Finally, a full-length hENT1 variant (residues 1-456, N48Q) with the glycosylation site eliminated was cloned into the pCAG plasmid for transient expression in human HEK293F cells. The choice of detergent proved to be vital for hENT1 extraction and purification. Following an exhaustive screening of detergents including DDM (n-dodecyl-β-D-maltoside), DM (n-decyl-β-D-maltoside), OG (n-octyl-β-D-glucoside), LMNG (2,2-Didecylpropane-1,3-bis-β-D-maltoside) and CHAPSO, the non-ionic detergent DDM appeared to be most effective in terms of solubilizing recombinant hENT1. The hENT1 protein was purified from the membrane fractions by nickel affinity chromatography and further fractionated by gel filtration. The purified hENT1 exhibited excellent solution behavior (Fig. 1B) and could be easily visualized on SDS-PAGE by coomassie blue staining (Fig. 1C). The peak fractions from gel filtration contain two co-migrating bands on the SDS-PAGE gel (Fig. 1C). Samples from both bands were confirmed to be hENT1 by mass spectrometry. hENT1 has a molecular weight of approximately 50.2-kDa, which is consistent with that of the lower band on the gel (Fig. 1C). The higher molecular weight band may correspond to the oligomeric form of hENT1. The analysis is supported by a recent report that hENT1 expressed in Sf9 insect cells contained an oligomeric form (Rehan and Jaakola, 2015).

### Transport activity of hENT1

**Figure 2:**
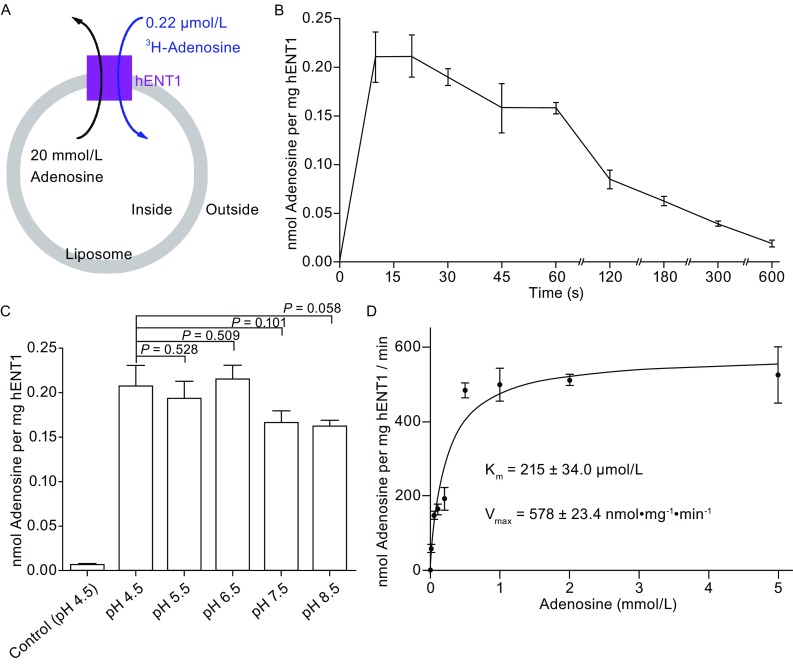
Transport activity of hENT1. (A) A schematic diagram of the counter flow assay. Uptake of ^3^H-labeled adenosine into the hENT1-incorporated liposome was monitored. (B) A time course analysis of the transport activity of hENT1. (C) The transport activity of hENT1 is pH-independent as measured in the liposome-based counter flow assay. P values between different pH values and pH 4.5 are indicated. Control (pH 4.5) refers to the condition where protein-free liposomes were used. (D) Determination of K_m_ and V_max_ for the transport of adenosine by hENT1. hENT1 proteoliposome was incubated in the presence of increasing concentrations of unlabeled adenosine. The K_m_ value was determined by non-linear regression analysis using GraphPad Prism version 5.0 software. Each data point represents the average of at least three independent experiments. The error bars represent standard deviation (SD)

All reported studies aimed at characterizing the ENTs transport mechanism relied exclusively on assays that were performed in native cell membranes (Ward et al., 2000; SenGupta and Unadkat, 2004; Endres and Unadkat, 2005; Visser et al., 2007; Paproski et al., 2008; Aseervatham et al., 2015). No homogeneous mammal ENTs protein has been used in any *in vitro* reconstituted assay. In the present investigation, we reconstituted the homogeneous, recombinant hENT1 protein into liposomes and established a proteoliposomes-based counter flow assay for the detection of substrate transport (Fig. 2A). In this assay, the tritium (^3^H)-labeled substrate adenosine was present in the outside buffer and its uptake into the liposomes was monitored (Fig. 2A). Compared to the protein-free liposomes or the liposomes with incorporation of a control membrane transporter GLUT3 (Deng et al., 2015), only hENT1-incorporated liposomes supported the uptake of ^3^H-adenosine (Fig. S1A).

A time course analysis of substrate uptake showed that adenosine was most rapidly transported into the liposomes within the initial 10 s and the accumulated amount of adenosine in the liposomes remained largely unchanged between 10 and 30 s (Fig. 2B). Due to the nature of the counter flow assay and the much higher concentrations of the unlabeled adenosine in the liposomes, the amount of ^3^H-adenosine in the liposomes gradually declined after 30 s (Fig. 2B). Nonetheless, the 30-second time point is more amenable to manipulation and allows acquisition of more accurate data. Therefore, except for the determination of V_max_, we used the 30-second time point for the qualitative characterization of hENT1. First, we investigated the pH dependence of hENT1 transport activity. The accumulated amount of ^3^H-adenosine in the liposomes remained largely pH-independent from pH 4.5 to 8.5 (Fig. 2C). This analysis suggests no critical role by Asp, Glu, or His residues in the transport mechanism of hENT1.

Next, we sought to determine the K_m_ and V_max_ of hENT1 transport at pH 6.5. Using varying concentrations of unlabeled adenosine and a constant concentration of ^3^H-adenosine outside the liposomes, we measured the amount of accumulated ^3^H-adenosine in the liposome at the 10-second time point. The transport rates were plotted against the concentrations of the substrate (unlabeled adenosine); the plot conformed to simple Michaelis-Menten kinetics and allowed determination of the K_m_ and V_max_ values (Fig. 2D). The calculated K_m_ value of 215 ± 34 µmol/L for the purified hENT1 is about half of that derived from hENT1 expressed in *Xenopus* oocytes (Aseervatham et al., 2015) (Fig. S1B), but 5-10 folds higher than those from hENT1 expressed in yeast and PK15 cell (Ward et al., 2000; Yao et al., 2001; Visser et al., 2005; Paproski et al., 2008) (Fig. S1B). Such differences can be attributed to a range of factors, including but not limiting to differences in lipid environment, surrounding protein factors, and quantification methods.

The inhibitors of ENTs modulate a variety of physiological processes by changing the extracellular adenosine concentrations and ENT inhibitors have a well-established role in the treatment of many diseases including cancer, HIV, and cardiovascular diseases (King et al., 2006; Jordheim et al., 2013). For example, the cytotoxic effect of the chemotherapeutic agent cladribine to the cultured acute lymphocytic leukaemia cells is enhanced by subsequent treatment with nitrobenzylmercaptopurine ribonucleoside (NBMPR) (Wright et al., 2000). Consistent with its therapeutic activity, NBMPR displays an inhibitory constant (IC_50_) of approximately 141 nM for hENT1 in the *in vitro* counter flow assay (Fig. S1C).

### Specific binding of adenosine and adenine to hENT1

**Figure 3:**
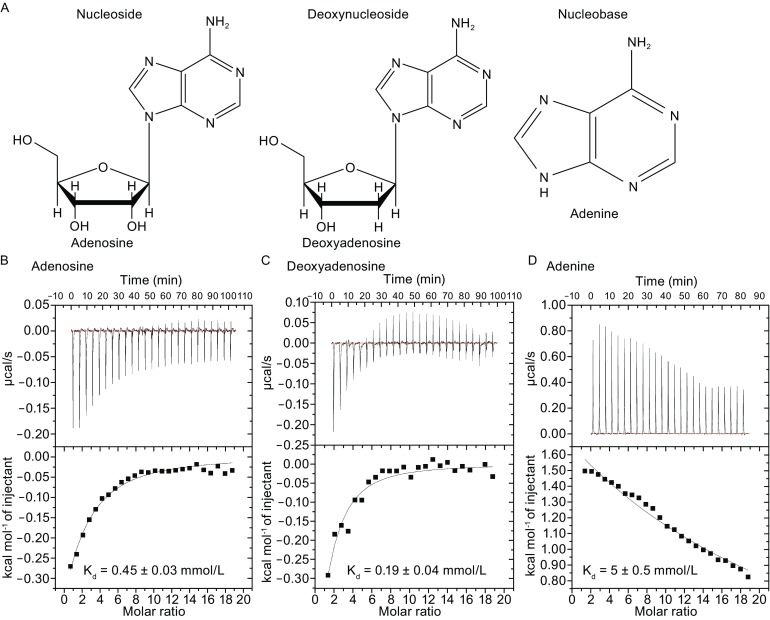
Determination of binding affinities between hENT1 and adenosine, deoxyadenosine, or adenine. (A) Chemical structures of adenosine, deoxyadenosine, and adenine. (B) Measurement of the binding affinity between hENT1 and adenosine by ITC. Curve fitting revealed a dissociation constant of 0.45 ± 0.03 mmol/L. The curve was fitted by Origin 7. (C) Measurement of the binding affinity between hENT1 and deoxyadenosine by ITC. (D) Measurement of the binding affinity between hENT1 and adenine by ITC

hENT1 and hENT2 are known to transport a broad range of purine and pyrimidine nucleosides (Ward et al., 2000; Baldwin et al., 2004; Boswell-Casteel and Hays, 2016; Young, 2016) (Fig. 3A and Fig. S2). In contrast to hENT2 that can efficiently transport nucleobases, hENT1 is thought to have much reduced transport ability for nucleobases (Yao et al., 2002; Yao et al., 2011). This functional property of hENT1 is likely related to its ability to bind the nucleosides versus the nucleobases. To assess this possibility, we used isothermal titration calorimetry (ITC) to determine the thermodynamic parameters of hENT1-ligand interactions in solution. In these experiments, concentrated substrates (5 mmol/L nucleosides or 10 mmol/L nucleobase) in the syringe were injected into the cell, where hENT1 was present at 0.1 mmol/L. The calculated dissociation constant (K_d_) between hENT1 and adenosine is approximately 0.45 ± 0.03 mmol/L, which is in good agreement with the calculated K_m_ value as determined in the proteoliposome-based counter flow assay (Fig. 2D and Fig. 3B). Intriguingly, hENT1 shows a slight preference for deoxyadenosine over adenosine, with a K_d_ of 0.19 ± 0.04 mmol/L (Fig. 3C). In sharp contrast to adenosine and deoxyadenosine, hENT1 binds the nucleobase adenine with a markedly lower dissociation constant of 5 ± 0.5 mmol/L (Fig. 3D). This finding suggests that the ribose moiety may be directly recognized by amino acids from hENT1.

The interactions between the nucleosides/nucleobases and hENT1 are highly specific. In the ITC assays, the purified hENT1 protein exhibited no detectable binding to the nucleoside guanosine, 5-methyuridine, uridine, or cytidine (Fig. S3A). hENT1 also failed to interact with the deoxynucleoside deoxyguanosine, thymidine, deoxyuridine, or deoxycytidine (Fig. S3B). Finally, hENT1 displayed no binding to the nucleobase guanine, thymine, uracil, or cytosine (Fig. S3C). Guanosine or deoxyguanosine has the same ribose (deoxyribose) backbone with adenosine or deoxyadenosine; but hENT1 exhibited no detectable binding to guanosine or deoxyguanosine in the ITC assays. These results suggest that the adenine base is specifically recognized by amino acids from hENT1. Taken together, our data demonstrate that hENT1 only specifically bind adenosine, deoxyadenosine, and to a lesser extent adenine while exhibiting no apparent binding to other nucleosides, deoxynucleosides, or nucleobases. These results indicate that both nucleobase and ribose/deoxyribose are involved in interactions with hENT1.

### Transport of nucleosides and nucleoside-derived drugs by hENT1

**Figure 4:**
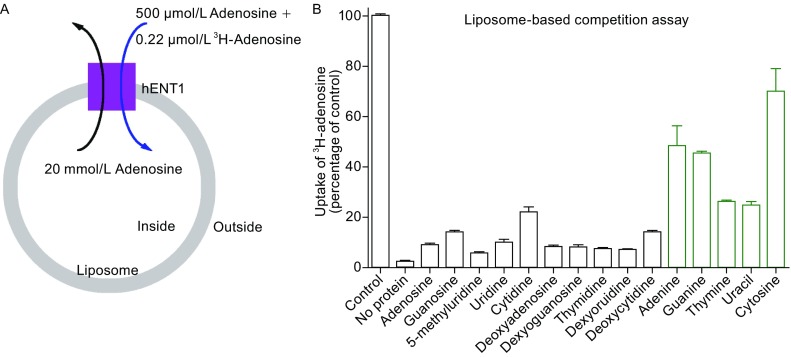
Substrate specificity of hENT1. (A) A schematic diagram of the competition assay. The proteoliposomes were preloaded with 20 mmol/L unlabeled adenosine, then diluted into 100 μL assay buffer containing 0.22 μmol/L ^3^H-adenosine and 500 μmol/L competitor - one of the listed nucleosides and nucleobases in panel B. (B) Transport activity for nucleosides, deoxynucleosides, and nucleobases. The transport of ^3^H-adenosine by recombinant hENT1 protein was examined in the proteoliposome-based counter flow assays in the presence of the indicated nucleosides, deoxynucleosides, and nucleobases. Control refers to the condition where no other substrate was added. No protein refers to the condition where protein-free liposomes were used. The black boxes represent the transport activity of nucleoside and deoxynucleoside for hENT1, whereas the green boxes represent the transport activity for nucleobase. Each data point represents the average of at least three independent experiments. The error bars represent standard deviation (SD)

All four hENT members can transport adenosine, but are distinguished functionally by their different transport capabilities for other nucleosides and nucleobases (Ward et al., 2000; Yao et al., 2002; Barnes et al., 2006; Govindarajan et al., 2009; Yao et al., 2011). We reconstituted a liposome-based competition assay to examine substrate selectivity by hENT1 (Fig. 4A). In this assay, concentrations of the unlabeled adenosine in the liposomes and ^3^H-adenosine in the buffer were kept constant, and 500 µmol/L nucleoside or nucleobase was added to the buffer. The transport activity of hENT1 for ^3^H-adenosine was more than 90 percent reduced by the presence of unlabeled adenosine, 5-methyluridine, uridine, deoxyadenosine, deoxyguanosine, thymidine, and deoxyuridine (Fig. 4B). Unlabeled guanosine, cytidine, and deoxycytidine also markedly decreased the transport of ^3^H-adenosine by hENT1 (Fig. 4B). In contrast, the nucleobases adenine, guanine, thymine, uracil, and cytosine were less efficient in the competition assay (Fig. 4B). Because these nucleosides and nucleobases exhibit no detectable binding to hENT1, they likely competed with ^3^H-adenosine for transport by hENT1, rather than for binding by hENT1. These results are consistent with previous conclusion that hENT1 robustly transports nucleosides and to a lesser extent nucleobases (Yao et al., 2011).

**Figure 5:**
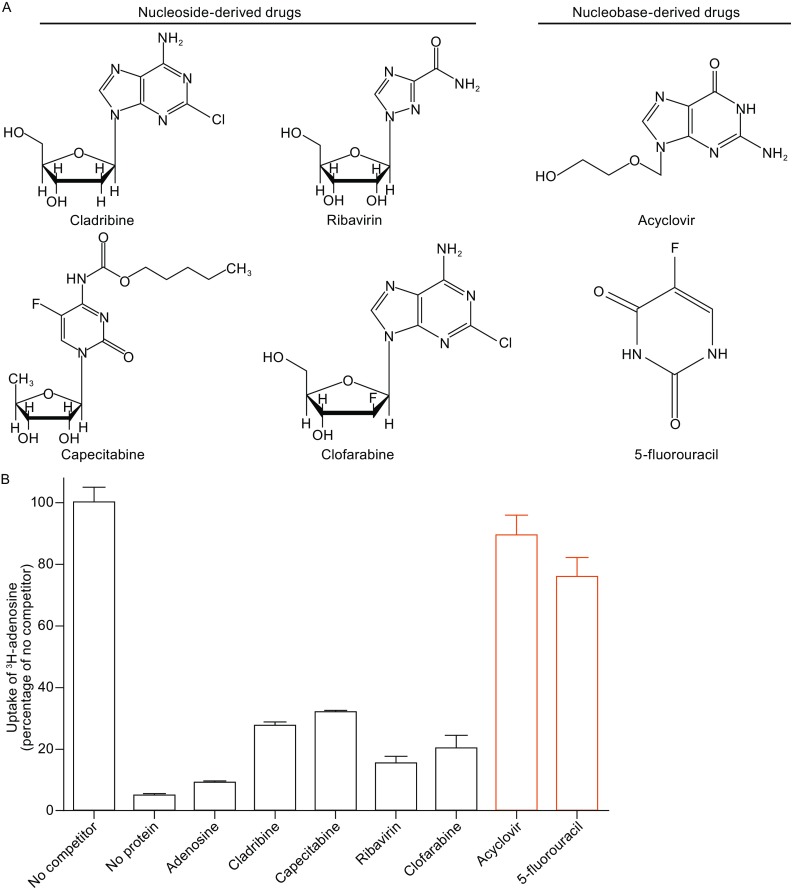
Nucleoside-derived drugs inhibit the transport activity of hENT1. (A) Chemical structures of representative nucleoside-derived and nucleobase-derived drugs. The drugs were used to treat cancer and virus infection in the clinic. (B) Transport activity of hENT1 for nucleoside-derived and nucleobase-derived drugs. No competitor refers to the condition where no other substrate was added. No protein refers to the condition where protein-free liposomes were used. The black boxes represent the transport activity of nucleoside-derived drugs for hENT1, whereas the orange boxes represent the transport activity for nucleobase-derived drugs. Each data point represents the average of at least three independent experiments. The error bars represent standard deviation (SD)

Nucleoside-derived and nucleobase-derived drugs are used in antiviral and anticancer therapies, and their interactions with ENTs have been extensively discussed (Baldwin et al., 2004; Jordheim and Dumontet, 2007; Jordheim et al., 2013; Young et al., 2013; Nordh et al., 2014; Catala et al., 2016). For example, capecitabine, clofarabine, and 5-fluorouracil have efficacy on a variety of solid tumors, and ribavirin and acyclovir are used to treat several forms of viral infection (Jordheim et al., 2013; Young et al., 2013) (Fig. 5A). In all cases, an essential step during treatment is for the drug to be efficiently imported into the cells. Presumably these nucleoside- and nucleobase-derived drugs are transported by ENTs. To investigate this scenario, we performed a similar liposome-based competition assay (Fig. 5B). Somewhat surprisingly, none of these drugs was able to compete as efficiently as adenosine. The nucleobase-derived drugs acyclovir and 5-fluorouracil failed completely to compete (Fig. 5B). These results strongly argue that, if hENT1 were the primary target for any of these drugs as widely suspected in the clinic, more potent drugs should be uncovered through chemical modification of existing ones. Presumably, drugs that retains high-affinity binding to hENT1 but cannot be transported by hENT1 may represent the best possible drug candidate. Most notably, elevated hENT1 expression is regarded as a therapeutic biomarker for pancreatic adenocarcinoma and breast cancer in the clinic (Nordh et al., 2014; Pastor-Anglada and Perez-Torras, 2015).

### Impact of hENT1 mutation on transport

**Figure 6:**
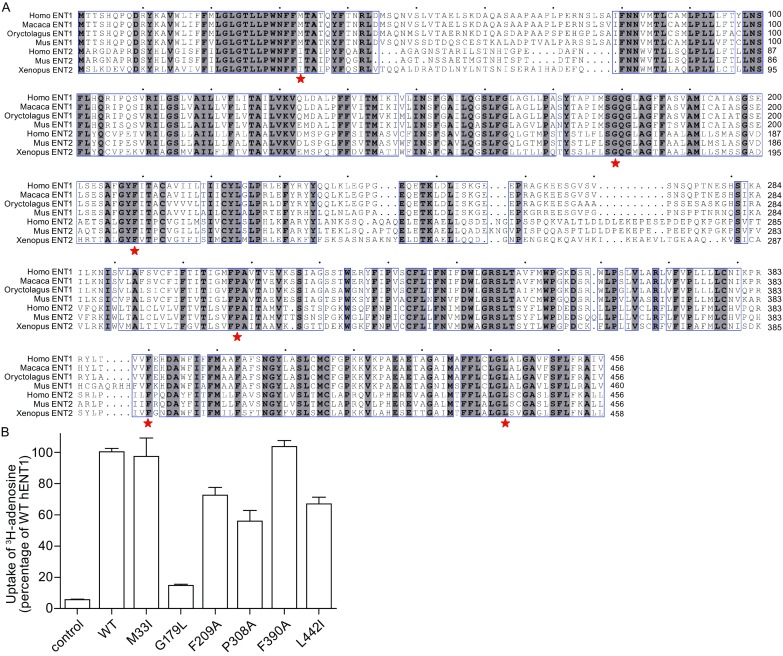
Effect of mutations on the transport activity of hENT1. (A) Sequence alignment among hENT1, three ENT1 orthologs from other species, hENT2, and two ENT2 orthologs from other species. Homo ENT1: *Homo sapiens*, GI: 118582262; Macaca ENT1: *Macaca mulatta*, GI: 383872534; Oryctolagus ENT1: *Oryctolagus cuniculus*, GI: 655841520; Mus ENT1: *Mus musculus*, GI: 312283707; Homo ENT2: *Homo sapiens*, GI: 38708299; Mus ENT2: *Mus musculus*, GI: 194248086; Xenolus ENT2: *Xenopus tropicalis*, GI: 62859387. Conserved residues are highlighted in gray. Six representative residues in panel B are indicated by red asterisks. (B) Transport activity of hENT1 variants. Six variants of hENT1 were generated and the proteins were individually purified to homogenous for transport assay. Control refers to the condition where protein-free liposomes were used. Each data point represents the average of at least three independent experiments. The error bars represent standard deviation (SD)

hENT1 and hENT2 exhibit high sequence identity (Fig. 6A). To identify functional residues, we mutated six conserved amino acids and individually purified the six hENT1 variants to homogeneity for the detection of their transport activity. Met33 of hENT1 was previously shown to be responsible for sensitivity to inhibitors such as dilazep and dipyridamole, but not NBMPR (Visser et al., 2002). In our assay, the mutation M33I had no effect on the transport activity of hENT1 (Fig. 6B). Gly179 of hENT1 was thought to be crucial for nucleoside transport and sensitive to inhibition by NBMPR (SenGupta et al., 2002). Consistent with the published data, the mutation of Gly179 to Leu (G179L) led to 90 percent reduction of the transport activity for adenosine (Fig. 6B). Four additional mutations, each involving a conserved hydrophobic residue, had different impacts on the transport activity of hENT1. No significant difference (*P* = 0.2469) was detected in the ability of adenosine transport for F390A (Fig. 6B). While compared to WT protein, mutations F209A (*P* < 0.01), P308A (*P* < 0.001), and L442I (*P* < 0.001) exhibited significantly reduced adenosine uptake activity (Fig. 6B). These results suggest that Phe209, Pro308 and Leu442 may have direct or indirect interactions with adenosine.

To verify the effect of these mutations on the transport activity of hENT1, a structural model for hENT1 was generated (Fig. S4). Among the mutated residues, Met33, Gly179, Pro308, and Leu442 are located in the putative transport path of hENT1 (Fig. S4), suggesting potential involvement in the substrate transport. Consistent with this observation, G179L, P308A, and L442I led to a significant decrease in adenosine transport (Fig. 6B). Met33 is responsible for the sensitivity to inhibitors (Visser et al., 2002), but not for the substrate transport (Fig. 6B). Phe209 is positioned on TM6 and F209A probably lead to the impairment of conformational flexibility required for transport activity (Fig. 6B and Fig. S4). Phe390 is far away from the transport path (Fig. S4), which is consistent with the functional assay in which F390A has no effect on the transport activity (Fig. 6B).

## Discussion

Despite growing interests in hENTs (Aseervatham et al., 2015; Rehan et al., 2015; Rehan and Jaakola, 2015), many of their biochemical properties such as substrate binding and transport mechanism are poorly understood. Biochemical characterization of hENTs has been limited by the inability to obtain recombinant protein with transport activity. Consequently, functional analysis of these transporters has been performed in native membranes (Ward et al., 2000; SenGupta and Unadkat, 2004; Endres and Unadkat, 2005; Visser et al., 2007; Paproski et al., 2008; Aseervatham et al., 2015; Rehan et al., 2015; Rehan and Jaakola, 2015), which is affected by other nucleoside transporters such as CNTs and multidrug resistance transporters. To date, only a putative ENT homologue FUN26 from *S. cerevisiae* was purified to homogeneity and incorporated into proteoliposome (Boswell-Casteel et al., 2014). In this study, we report the successful expression of hENT1 in human HEK293F cells. The recombinant protein was purified to homogeneity and subjected to functional characterization in the liposome-based counter flow assay and isothermal titration calorimetry. To our knowledge, such effort represents the first of its kind for any mammalian ENT transporter.

Although hENT1 binds to adenosine and deoxyadenosine, it fails to interact with other nucleosides or deoxynucleosides (Fig. S3). This result is a bit surprising, suggesting high specificity for the adenine base. Despite the binding preferences, adenosine transport by hENT1 is competitively inhibited by these nucleosides and deoxynucleosides (Fig. 4B). Previous studies showed that these nucleosides and deoxynucleosides could be transported by hENT1 (Vickers et al., 1999; Ward et al., 2000; Yao et al., 2002; Baldwin et al., 2004; Young et al., 2013). Thus, these nucleosides and deoxynucleosides competed with adenosine for transport by hENT1, hence reducing its activity for adenosine.

The inhibitor NBMPR exhibited an IC_50_ of about 141 nM for the transport activity of hENT1 (Fig. S1C). Given the similarity in their chemical structures, we suspected that NBMPR directly competed with adenosine for binding to hENT1. Surprisingly, however, no detectable binding was observed between NBMPR and hENT1 by ITC (data not shown). This result strongly argues that NBMPR, similar to other nucleosides, may simply compete with adenosine for transport by hENT1.

Taken together, we report successful purification and biochemical characterization of hENT1 *in vitro* functional assay. Results reported in this manuscript represent improve our understanding on the transport properties of hENT1. Obviously, such biochemical data should be ideally reconciled by structural information, which is ostensibly absent for any mammalian ENT member. Nonetheless, purification of hENT1 in a functional form constitutes a major step towards its eventual crystallization and structure determination.

## Materials and Methods

### Protein preparation

The cDNA of wild-type (WT) human ENT1 (hENT1) with a single N48Q mutation was cloned into the pCAG vector. Endo-free plasmid pre-mixed with linear polyethylenimines (PEIs) (Polysciences) (1:3, w/w) was transfected into HEK293F cells (Invitrogen) when the density reached 2 × 10^6^ cells/mL. For each experiment, approximately 1 mg plasmids were pre-mixed with 3 mg PEIs in 50 mL fresh SMM 293T-I medium (Sino Biological Inc.) for 15–30 min before transfection. For transfection, the 50 mL mixture was added to one Liter cell culture and incubated for 30 min. Cells were cultured in the incubator shaker (INFORS HT, Multitron Pro) at 37°C. After growth for 72 h, the transfected cells were harvested, resuspended in lysis buffer containing 25 mmol/L Tris-HCl, pH 8.0, 150 mmol/L NaCl, and protease inhibitor cocktails (Amresco), and disrupted by sonication on ice. Cell membrane was collected by ultracentrifugation at 150,000 *g* for 1 h. The membrane pellet was resuspended in the lysis buffer and incubated for 1.5 h with 2.0% (w/v) dodecyl-β-d-maltopyranoside (DDM, Anatrace) at 4°C. After another ultracentrifugation step at 150,000 *g* for 30 min, the supernatant was loaded onto Ni^2+^-nitrilotriacetate affinity resin (Ni-NTA; Qiagen), incubated at 4°C for 15 min and washed with buffer containing 25 mmol/L Tris-HCl, pH 8.0, 150 mmol/L NaCl, 30 mmol/L imidazole, and 0.02% DDM. The target protein was eluted from the affinity resin with buffer containing 25 mmol/L Tris-HCl, pH 8.0, 150 mmol/L NaCl, 250 mmol/L imidazole, and 0.02% DDM. The eluted solution was concentrated to about 5 mg/mL before further purification by gel filtration (Superdex-200, GE Healthcare) in buffer containing 0.02% DDM, 30 mmol/L HEPES, pH 7.4, and 150 mmol/L NaCl. The peak fractions were collected, flash-frozen in liquid nitrogen and stored at −80°C for later use.

### Preparation of liposomes and proteoliposomes

50 mg/mL *E.coli* polar lipids (Avanti Polar Lipids, Inc.) were dissolved in chloroform and methanol mixture (3:1, v/v), the organic solvent was removed by nitrogen stream. The remained lipid layer was resuspended to 20 mg/mL in assay buffer containing 40 mmol/L MES, pH 6.5, 40 mmol/L KCl, and 2 mmol/L MgSO_4_. After fast frozen and thawed for 10 cycles, the mixture was extruded through 0.4 μm membrane filter (Whatman). 1% *n*-octyl-β-d-glucoside (β-OG, Anatrace), 20 mmol/L adenosine, and the protein hENT1 or GLUT3 (a glucose transporter) was then added into the mixture (protein: lipid = 1:100, w/w). For blank control, equal volume of the same buffer used in the final step of protein purification was added. After incubation at 4°C for 1 h, β-OG was removed by incubation with 320 mg/mL Bio-Beads SM2 (Bio-Rad) overnight. After separation from detergent-absorbed beads, the mixture was repeatedly frozen and thawed for five cycles. The proteoliposomes were extruded through 0.4 μm membrane filter again and collected by an ultracentrifugation step at 100,000 *g* for 1 h. The collected proteoliposomes were washed twice with the cold assay buffer. The proteoliposomes were on ice and resuspended in cold assay buffer before use with a final concentration of 100 mg/mL.

### Liposome-based counter flow assay

All counter flow assays were performed at room temperature. For each assay, 2 μL of prepared proteoliposomes as described above was added into 98 μL of the assay buffer containing 0.5 μL of ^3^H-adenosine (23 Ci mmol^−1^, Moravek Chemicals) to start reaction. The final concentration of external ^3^H-adenosine was 0.22 μmol/L. Each reaction lasted 30 s, if not otherwise indicated, and was stopped by rapidly filtering the reaction solution through a 0.22-μm GSTF filter (Millipore). The filter membrane was washed with 2 mL assay buffer and solubilized with Optiphase HISAFE 3 (PerkinElmer), used for liquid scintillation counting. All experiments were repeated at least three times.

### Isothermal titration calorimetry

MicroCal iTC200 (GE Healthcare) was used to measure the binding affinities between hENT1 and substrates at 22°C. The hENT1 protein was prepared in a buffer containing 0.02% DDM, 30 mmol/L HEPES pH 7.4, and 150 mmol/L NaCl. Substrates were prepared in the same buffer, except that 1% DMSO or ethanol was used to help dissolve the substrates. During a titration experiment, the hENT1 protein concentration was 0.1 mmol/L in the cell, while substrates in the syringe were 5 mmol/L for nucleosides or deoxynucleosides, and 10 mmol/L for nucleobases. Data was fitted by software Origin 7.0 (MicroCal).

### Structural modelling of human ENT1 (hENT1)

A hENT1 structure model was constructed using RaptorX web server (Wang and Xu, 2013; Ma et al., 2015; Wang et al., 2016) based on contact map prediction. 1610 sequence homologs were used for multi-sequence alignment (MSA). The contact information extracting from MSA was considered on high confidence level for conserved regions. The hENT1 model appears to contain eleven transmembrane segments in general as reported.

## Electronic supplementary material

Below is the link to the electronic supplementary material.

Supplementary material 1 (PDF 4492 kb)
