# Supplementary material for: Functional characterization of human equilibrative nucleoside transporter 1
Source: Protein Cell. 2016 Dec 19;8(4):284–95. doi: 10.1007/s13238-016-0350-x (PMC5359181; doi:10.1007/s13238-016-0350-x)
Supplement: Supplementary file 1 — Supplementary material 1 (PDF 4492 kb) [file 13238_2016_350_MOESM1_ESM.pdf]

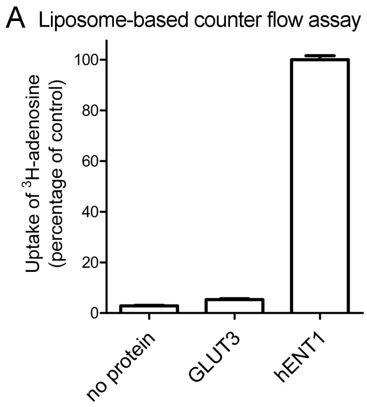

B

	K_m (μM)	System
hENT1	450 ± 50	oocyte cell[α]
	44 ± 7.5	yeast cell[β]
	19 ± 2	yeast cell[γ]
	17 ± 0.8	yeast cell[σ]
	40 ± 4	PK15 cell[ω]

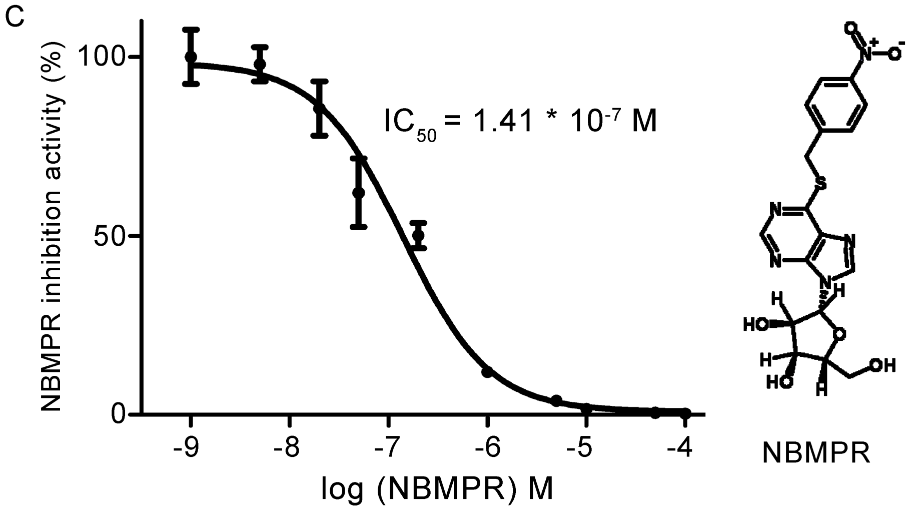

Figure S1. Transport activity of hENT1. (A) hENT1, but not the control membrane transporter GLUT3, efficiently transported adenosine. No protein refers to the condition where protein-free liposomes were used. (B) Reported K_m values for hENT1. The K_m values of hENT1 for adenosine were previously reported in studies using *Xenopus* oocyte, yeast, and PK15 cell. [α]Study published in ref. (Aseervatham et al., 2015), [β]Study published in ref. (Endres et al., 2004), [γ]Study published in ref. (Visser et al., 2007), [σ]Study published in ref. (Visser et al., 2005), and [ω]Study published in ref. (Ward et al., 2000). (C) NBMPR inhibits the transport activity of hENT1 *in vitro* in a dose-dependent manner. Uptake of ^{3}H-adenosine into the hENT1-incorporated liposomes is subject to competition by the addition of increasing concentrations of the inhibitor NBMPR.

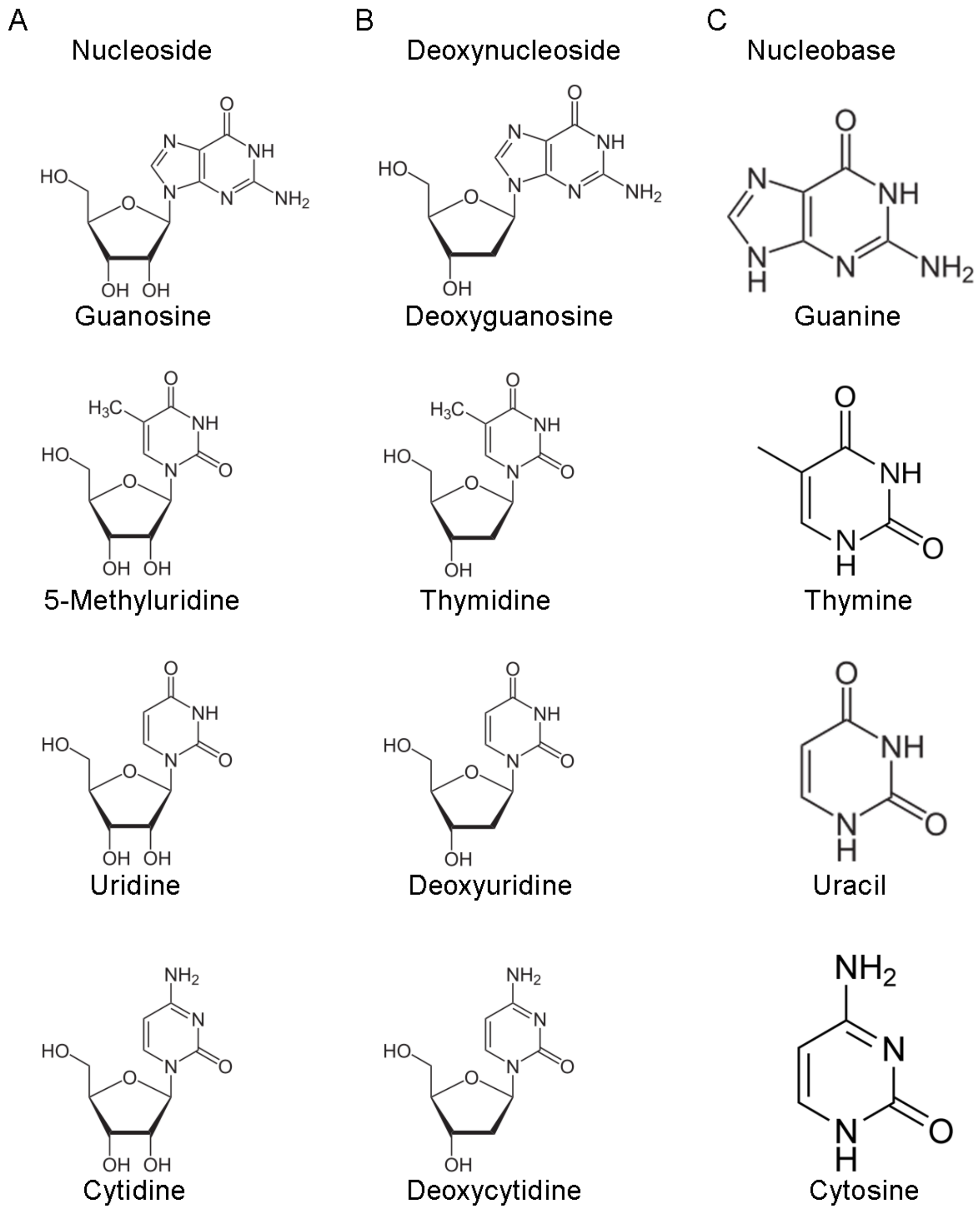

Figure S2. Chemical structures of representative nucleosides and nucleobases. Nucleobases (C) are nitrogen-containing biological compounds, which link a 5-carbon sugar, either ribose or deoxyribose to form nucleosides (A) or deoxynucleosides (B).

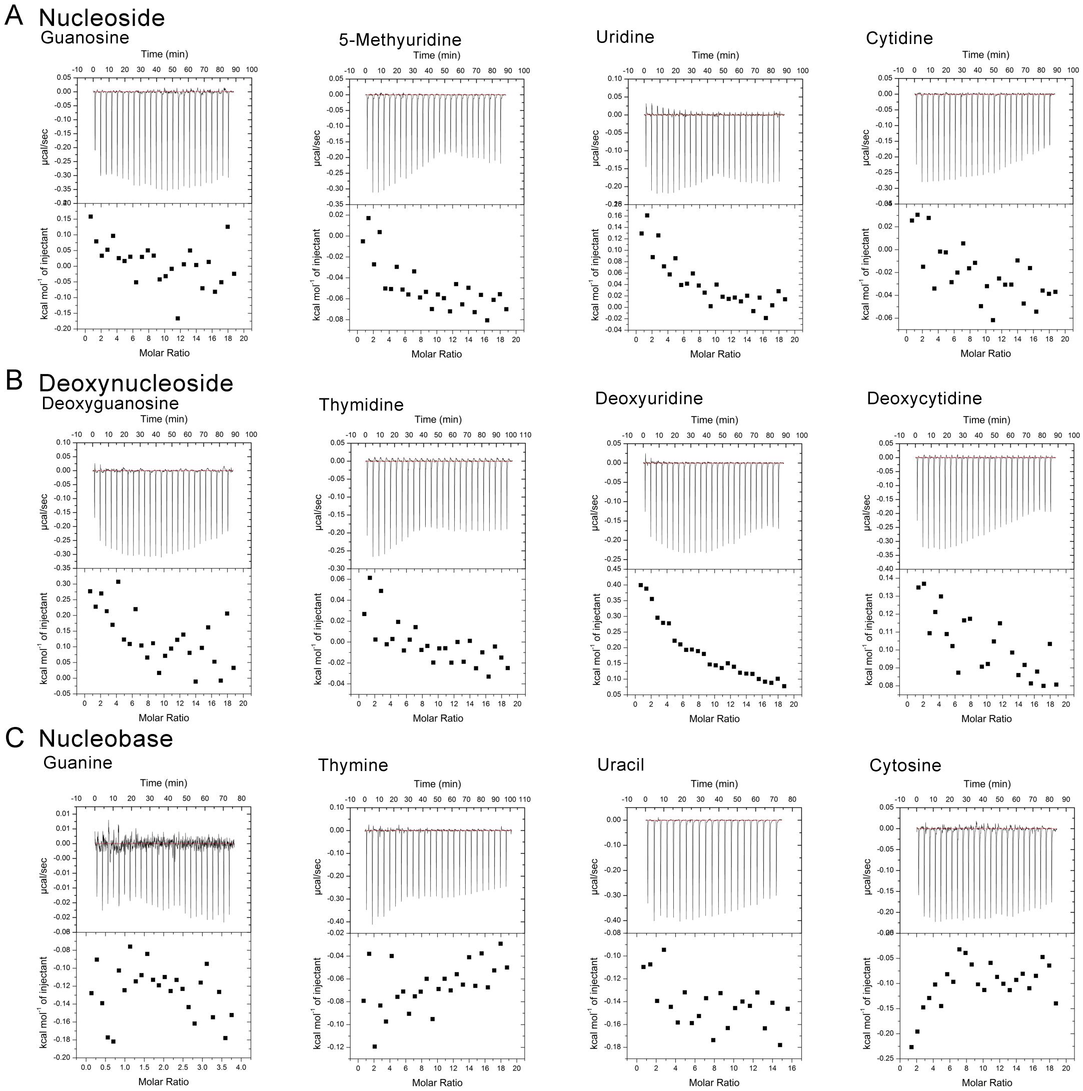

Figure S3. ITC analyses of nucleoside and nucleobase binding by hENT1. (A) None of the nucleosides examined exhibited detectable binding to hENT1 by isothermal titration calorimetry (ITC). Shown here from left to right are the ITC data for guanosine, 5-methyluridine, uridine, and cytidine. The ITC data for each nucleoside includes the original titration isotherm curves (upper panel) and the processed data points (lower panel). (B) None of the deoxynucleosides examined exhibited detectable binding to hENT1 by isothermal titration calorimetry (ITC). (C) None of the nucleobases examined exhibited detectable binding to hENT1 by isothermal titration calorimetry (ITC).

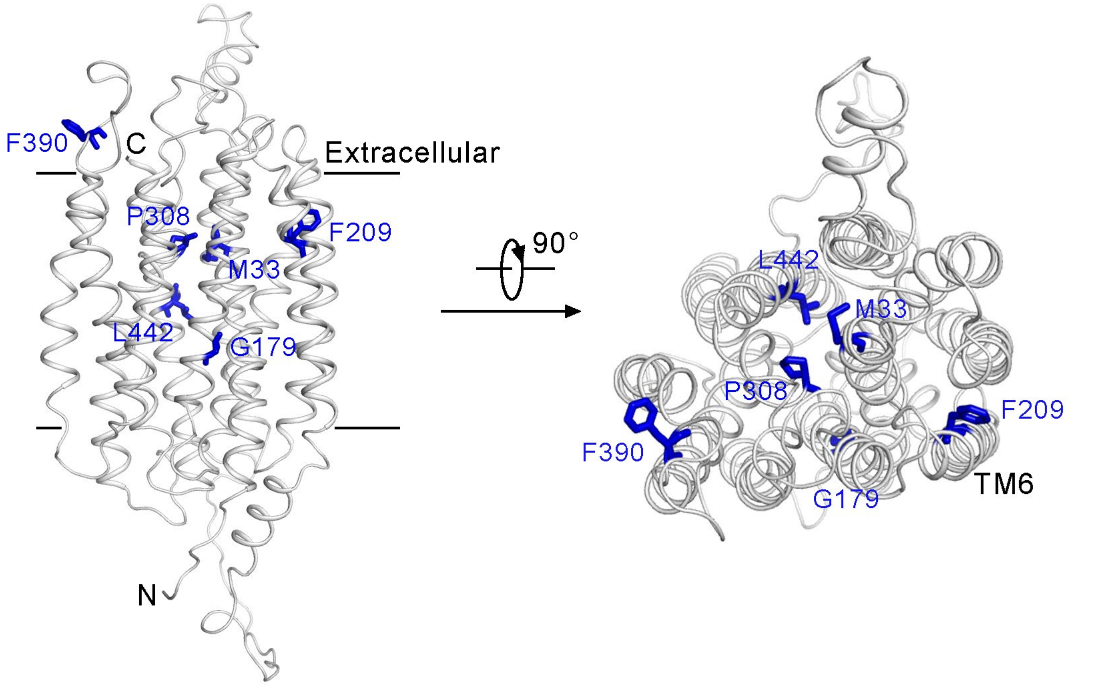

Figure S4. The structural model of hENT1. The structural model of hENT1 was generated as described in Materials and Methods. Two perpendicular views of hENT1 are shown by cartoon. Six representative residues in hENT1 are showed by sticks and labelled blue.

Supplementary References

Aseervatham, J., Tran, L., Machaca, K., and Boudker, O. (2015). The Role of Flexible Loops in Folding, Trafficking and Activity of Equilibrative Nucleoside Transporters. PLoS One 10, e0136779.

Endres, C.J., Sengupta, D.J., and Unadkat, J.D. (2004). Mutation of leucine-92 selectively reduces the apparent affinity of inosine, guanosine, NBMPR [S-6-(4-nitrobenzyl)-mercaptopurine riboside] and dilazep for the human equilibrative nucleoside transporter, hENT1. Biochem J 380, 131-137.

Visser, F., Sun, L., Damaraju, V., Tackaberry, T., Peng, Y., Robins, M.J., Baldwin, S.A., Young, J.D., and Cass, C.E. (2007). Residues 334 and 338 in transmembrane segment 8 of human equilibrative nucleoside transporter 1 are important determinants of inhibitor sensitivity, protein folding, and catalytic turnover. J Biol Chem 282, 14148-14157.

Visser, F., Zhang, J., Raborn, R.T., Baldwin, S.A., Young, J.D., and Cass, C.E. (2005). Residue 33 of human equilibrative nucleoside transporter 2 is a functionally important component of both the dipyridamole and nucleoside binding sites. Mol Pharmacol 67, 1291-1298.

Ward, J.L., Sherali, A., Mo, Z.P., and Tse, C.M. (2000). Kinetic and pharmacological properties of cloned human equilibrative nucleoside transporters, ENT1 and ENT2, stably expressed in nucleoside transporter-deficient PK15 cells. Ent2 exhibits a low affinity for guanosine and cytidine but a high affinity for inosine. J Biol Chem 275, 8375-8381.
